# Decreased GABA receptor in the cerebral cortex of epileptic rats: effect of *Bacopa monnieri *and Bacoside-A

**DOI:** 10.1186/1423-0127-19-25

**Published:** 2012-02-24

**Authors:** Jobin Mathew, Savitha Balakrishnan, Sherin Antony, Pretty Mary Abraham, CS Paulose

**Affiliations:** 1 Department of Zoology, CMS College Kottayam, Kerala-686 001, India; 2 Molecular Neurobiology and Cell Biology Unit, Centre for Neuroscience, Department of Biotechnology, Cochin University of Science and Technology, Cochin-682 022, Kerala, India

**Keywords:** Epilepsy, *Bacopa monnieri*, Bacoside-A, Pilocarpine, Carbamazepine

## Abstract

**Abstact:**

**Background**

Gamma amino butyric acid (GABA), the principal inhibitory neurotransmitter in the cerebral cortex, maintains the inhibitory tones that counter balances neuronal excitation. When this balance is perturbed, seizures may ensue.

**Methods**

In the present study, alterations of the general GABA, GABA_A _and GABA_B _receptors in the cerebral cortex of the epileptic rat and the therapeutic application of *Bacopa monnieri *were investigated.

**Results**

Scatchard analysis of [^3^H]GABA, [^3^H]bicuculline and [^3^H]baclofen in the cerebral cortex of the epileptic rat showed significant decrease in B_max _(P < 0.001) compared to control. Real Time PCR amplification of GABA receptor subunits such as GABA_Aά1_, GABA_Aγ_, GABA_Aδ_, GABA_B _and GAD where down regulated (P < 0.001) in epileptic rats. GABA_Aά5 _subunit and Cyclic AMP responsible element binding protein were up regulated. Confocal imaging study confirmed the decreased GABA receptors in epileptic rats. Epileptic rats have deficit in radial arm and Y maze performance.

**Conclusions**

*Bacopa monnieri *and Bacoside-A treatment reverses epilepsy associated changes to near control suggesting that decreased GABA receptors in the cerebral cortex have an important role in epileptic occurrence; *Bacopa monnieri *and Bacoside-A have therapeutic application in epilepsy management.

## Background

GABA is formed within GABAergic axon terminals and released into the synapse, where it acts at one of two types of receptor GABA_A _which controls chloride entry into the cell, and GABA_B_, which increases potassium conductance, decreases calcium entry, and inhibits the presynaptic release of other neurotransmitters [1,2]. Temporal lobe epilepsy (TLE) is considered the most common epileptic syndrome and it is estimated that approximately 80% of patients with partial seizures have temporal lobe epilepsy [3]. The effect of pilocarpine treatment, which is characterized by generalized convulsive status epilepticus in rodents, well represents the characteristic neuropathological findings in the brain regions of the patients with TLE epilepsy [4]. Imbalance between excitatory and inhibitory synaptic transmission in key brain areas are implicated in the pathophysiology of TLE, in which there is a decrease in the GABA mediated inhibition. TLE seizures reflect excess excitation, which result from local inhibitory circuit dysfunction. The existence of multiple GABA_A _receptor subtypes differing in subunit composition, localization and functional properties underlies their role for fine-tuning of neuronal circuits and genesis of network oscillations. The differential regulation of GABA_A _receptor subtypes represents a major facet of homeostatic synaptic plasticity and contributes to the excitation/inhibition (E/I) balance under physiological conditions and upon pathological challenges [5]. Glutamate decarboxylase (GAD) is an enzyme that catalyzes the decarboxylation of glutamate to GABA. GAD is the rate limiting enzyme of GABA synthesis and it is used as a marker for GABAergic activity [6]. cAMP response element-binding protein (CREB) is a transcription factor that has been implicated in the activation of protein synthesis required for long-term memory and seizure formation [7]. Findings shows that GABA_B _receptor subunit gene expression in hippocampal neurons is mediated through the CREB by binding to unique cAMP response elements in the alternative promoter regions [8]. Radial arm maze is a means to examine the neural systems that are involved in memory and the influence of pharmacological compounds on memory [9,10]. Y-maze used to evaluate the spatial learning by the different animal models of the rats [11]

The potential for antiepileptic drugs to negatively impact cognitive abilities is of significant concern because they are the major therapeutic modality for control of seizures. An increased risk for cognitive deficits has been noted in patients with temporal lobe seizures. Moreover, many of the anticonvulsant drugs presently used for treating epilepsy cannot prevent neurodegeneration but rather contribute towards these cognitive deficits [12,13]. The available anti-epileptic drugs are not curative since they mostly treat the symptoms of the disease and render little help to alleviate its cause. This has instilled a renewed interest in traditionally used herbal drugs and formulations, which are safe in prolonged usage for the management of epilepsy.

*Bacopa monnieri *is well known for its neuropharmacological effects. It is currently recognized as being effective in the treatment of mental illness and epilepsy [14]. Treatments with *Bacopa monnieri *extract [15] have enhanced learning ability. Cognition-facilitating effect was due to two active saponins, bacosides A and B present in the ethanol extract [16]. These active principles, apart from facilitating learning and memory in normal rats, inhibited the amnesic effects of scopolamine, electroshock and immobilization stress. Crude plant extract or bacosides have also shown anxiolytic effects, antidepressant activity, anticonvulsive action and antioxidant activity [17]. In vitro studies using *Bacopa monnieri *have been found to inhibit free radical formation and DNA damage in a dose dependent manner [14]. But so far there are very few studies reporting the role of *Bacopa monnieri *treatment on the functional regulation of GABA receptors. In this study, we investigated the anti-epileptic effect of *Bacopa monnieri *on the GABAergic receptor binding, gene expression in the cerebral cortex and spatial learning and memory in epileptic rats.

## Methods

### Chemicals Used in the Study

Biochemicals used in the present study were purchased from Sigma Chemical Co., St. Louis, USA. All other reagents were of analytical grade purchased locally [^3^H]GABA [^3^H]bicuculline and [^3^H]baclofen were purchased from NEN life sciences products Inc., Boston, U.S.A.

### Animals

Adult Wistar rats of 250-300 g body weight purchased from Amrita Institue of Medical Sciences, Cochin and Kerala Agriculture University, were used for all experiments. They were housed in separate cages under 12 hrs light and 12 hrs dark periods and were maintained on standard food pellets and water *adlibitum*. All animal care and procedures were taken in accordance with the Institutional, National Institute of Health and CPESCA guidelines.

### Plant material and Preparation of extract

Specimen of *Bacopa monnieri *(L.) Pennel were collected from Cochin University area and were taxonomically identified and authenticated by Mr. K. P. Joseph, Head, Dept. of Botany (retd.), St. Peter's College, Kollenchery and voucher specimens was deposited at a herbarium (No: MNCB3) of Centre for Neuroscience, Cochin University of Science and Technology, Cochin, Kerala, India. Fresh, whole *Bacopa monnieri *plant were collected and washed. Leaves, roots and stems of *Bacopa monnieri *plant were cut into small pieces and dried in shade. 100 g fresh plant dried in shade yielded approximately 15 g powder. Homogenate was extracted at required concentration (300 mg fresh plant/kg body weight) by dissolving 450 mg of dried powder in 80 ml distilled water and used for the study [18,19].

### Induction of Epilepsy in adult rats

Adult male Wistar rats, weighing 250 to 300 g, were housed for 1 to 2 weeks before experiments were performed. Epilepsy was induced by injecting rats with pilocarpine (350 mg/kg body weight i.p.), preceded by 30 min with atropine (1 mg/kg body weight i.p.) to reduce peripheral pilocarpine effects. Within 20 to 40 min after the pilocarpine injection, essentially all the animals developed status epilepticus (SE). Control animals were given saline injection. Behavioural observation continued for 5 hrs after pilocarpine injection. SE was allowed to continue for 1 hr and then control and experimental animals were treated with diazepam (4 mg/kg body weight i.p.). Animals recovered from this initial treatment within 2 to 3 days and were observed for the next 3 weeks. 24 days after pilocarpine treatment, the rats were continuously video monitored for 72 hrs. The behaviour and seizures were captured with a CCD camera and a Pinnacle PCTV capturing software card. One trained technician, blind to all experimental conditions, viewed all videos. Seizure activity was rated according to Racine Scale [20]. Seizures were assessed by viewing behavioural postures (i.e. lordosis, straight tail, jumping/running, forelimb clonus and/or rearing) during observation of the videos. Experimental rats which showed recurrent seizures were used for the further experiments. Experimental rats were divided into five groups: 1) Control (C), 2) Epileptic (E), 3) Epileptic rats treated with *Bacopa monnieri *(E+B), 4) Epileptic rats treated with bacoside-A (E+D), 5) Epileptic rats treated with carbamazepine (E+C). *Bacopa monnieri *treated rats were given extract of *Bacopa monnieri *orally in the dosage 300 mg/kg body weight/day for 15 days. Carbamazepine- a standard drug used for the treatment of epilepsy was given orally in the dosage 150 mg/kg body weight/day for 15 days. Bacoside-A was given orally in the dosage 150 mg/kg body weight/day for 15 days. After the treatment the rats were sacrificed and the tissues were stored in -80°C.

### Protein Determination

Protein was measured by the method of Lowry et al [21] using bovine serum albumin as standard. The intensity of the purple blue colour formed was proportional to the amount of protein, which was read in a spectrophotometer at 660 nm.

### GABA Receptor Binding Assay

[^3^H]GABA binding to GABA, [^3^H]bicuculline to GABA_A _and [^3^H]baclofen binding to GABA_B _receptors were assayed in triton x-100 treated synaptic membranes according to the procedure of Kurioka et al [22]. Crude synaptic membrane was prepared using sodium-free 10 mM Tris buffer (P^H^7.4). Each assay tube contained a protein concentration of 100 mg. In saturation binding experiments, 5-40 nM of [^3^H] GABA incubated with and without excess of unlabelled GABA (100 μM). The incubation was continued for 20 min at 40°C and terminated by centrifugation at 35000 xg for 20 min. [^3^H]GABA in the pellet was determined by liquid scintillation spectrometry. Specific binding was determined by subtracting non-specific binding from the total binding.

### Real-Time Polymerase Chain Reaction

Total RNA was isolated from the cerebral cortex of control and experimental rats using Tri reagent. RNA was reverse transcribed using ABI PRISM cDNA Archive kit. 20 μl of the reaction mixture contained 0.2 μg total RNA, 10X RT buffer, 25X dNTP mixture, 10X Random primers, MultiScribe RT (50 U/μl) and RNAase free water. The reactions were carried out at 25°C for 10 minutes and 37°C for 2 hours using an Eppendorf Personal Cycler. Real-Time PCR assays were performed using specific primer and fluorescently labeled Taq probe in an ABI 7300 Real-Time PCR instrument (Applied Biosystems). The TaqMan reaction mixture of 20 μl contained 25 ng of total RNA-derived cDNAs, 200 nM each of the forward primer, reverse primer and TaqMan probe for specific gene, endogenous control, β-actin and 12.5 μl of TaqMan 2X Universal PCR Mastermix. The thermocycling profile conditions used were: 50°C - 2 minutes - Activation; 95°C - 10 minutes - Initial Denaturation; followed by 40 cycles of 95°C - 15 seconds - Denaturation and 60°C-1 minute - Annealing. The ΔΔCT method of relative quantification was used to determine the fold change in expression. This was done by first normalizing the resulting threshold cycle (CT) values of the target mRNAs to the CT values of the internal control β-actin in the same samples (ΔCT = CT_Target _- CT_β-actin_). It was further normalized with the control (ΔΔCT = ΔCT - CT _Control_). The fold change in expression was then obtained as (2^-ΔΔCT^) and expressed as log 2^-ΔΔCT^.

The sequences of the probe were

GABA_Aά1 _:- ATTTGGGAGCTATGCTTATACAAGA

GABA_Aά5 _:- CAGCACCAGCACAGGTGAATATACA

GABA_Aγ3 _:- GCATGCTCGGTCCAGGAGGGTGGAA

GABA_Aδ _:- CCGCACCATGGCGCCAGAGCAATGA

GAD65:- AGTCATTACAAATCT TGCCC

The sequences of the primers were

GABA_Aά1 5' ACA AGA AGC CAG AGA ACA AGC CAG 3'_

_5' GAG GTC TAC TGG TAA GCT CTA CCA 3'_

GABA_Aά5 5' TGA GAT GGC CAC ATC AGA AGC AGT 3'_

_5' TCA TGG GAG GCT GGA GTT TAG TTC 3'_

GABA_Aγ3 5' CAG AGA CAG GAA GCT GAA AAG CAA 3'_

_5' CGA AGT GAT TAT ATT GGA CTA AGC 3'_

GABA_Aδ 5' TGT GAG CAA CCG GAA ACC AAG CAA-3'_

_5' CGT GTG ATT CAG CGA ATA AGA CCC-3'_

GAD65 _5'GCCCAGGCTCATCGCATTCA-3'_

_5'AGTCATTACAAATCT TGCCC-3'_

### Linear regression analysis for Scatchard plots

The data were analysed according to Scatchard [23]. The specific binding was determined by subtracting non-specific binding from the total. The binding parameters, maximal binding (B_max_) and equilibrium dissociation constant (K_d_), were derived by linear regression analysis by plotting the specific binding of the radioligand on X-axis and bound/free on Y-axis. The maximal binding is a measure of the total number of receptors present in the tissue and the equilibrium dissociation constant is the measure of the affinity of the receptors for the radioligand. K_d _is inversely related to receptor affinity.

### Radial Maze Test

Radial maze behavioral testing was conducted under normal room lighting and utilized an eight armed radial maze elevated 100 cm from the floor. Each arm of the maze (11.5 cm wide) extended 68.5 cm from an octagonally shaped central platform (40 cm across). Black Plexiglas walls (11.5 cm high) were present only for the first 20 cm of each arm to prevent the rat crossing from one to another without returning to the central platform. Circular food wells (1.3 cm deep, 3.2 cm diameter) were located 2.5 cm from the end of each arm. The maze was centered in an enclosed room where lighting and spatial cues (e.g., posters, door, and boxes) remained constant throughout the course of the experiment. Arms were baited by placing one raisin in each food well.

Rats were placed on the maze 3 days prior to the start of formal acquisition testing in order to habituate them to the apparatus. On the first day of habituation, 4 food pellets were scattered along the length of each arm. The rats were then systematically confined to each arm for 1 min to ensure their exposure to the entire maze. On the second day of habituation, the previous day's procedure was repeated except that the animals were not confined to each arm following 5 min of exploration. On the third day, one food pellet was placed in the food well at the end of each arm and a second was placed halfway down each arm. Once the rats were habituated to the maze, testing began. Trials began by placing a single rat in the center of the maze facing away from the experimenter. The trial ended when the rat had obtained all 4 pellets or 5 min had elapsed, whichever occurred first. Rats were run until they achieved criterion performance for task acquisition. Criterion was attained when the rat collected 3 out of the 4 food pellets within their first 4 arm entries within a trial (while still completing the trial) with this level of performance being maintained for 5 consecutive criterion performance. The number of trials up to and including the last of these 5 criterion performance formed the "number of trials to criterion" measure. Experimental subjects were tested under blind conditions. The time of testing was consistent from day to day for each subject but testing of the various treatment groups was distributed randomly throughout the day.

### Y-Maze Test

The Y-maze was made of grey wood, covered with black paper, and consisted of three arms with an angle of 120°between each of the arms. Each arm was 8 cm width ×30 cm length ×15 cm height. The three identical arms were randomly designated: Start arm, in which the mouse started to explore (always open); Novel arm, which was blocked at the 1st trial, but open at the 2nd trial; and the other arm (always open). The maze was placed in a separate room with enough light. The floor of the maze was covered with sawdust, which was mixed after each individual trial in order to eliminate olfactory stimuli. Visual cues were placed on the walls of the maze.

The Y-maze test consisted of two trials separated by an inter-trial interval (ITI). The first trial (training) was 10 min duration and allowed the mouse to explore only two arms (start arm and the other arm) of the maze, with the third arm (novel arm) blocked. After a 1 h ITI [24], the second trial (retention) was conducted, during which all three arms were accessible and novelty vs. familiarity was analyzed through comparing behavior in all three arms. For the second trial, the mouse was placed back in the maze in the same starting arm, with free access to all three arms for 5 min. The time spent in each arm were analyzed data were expressed as percentage of performance in all three arms during the 5-minutes of test [25].

### Confocal imaging

Control and experimental rats were deeply anesthetized with ether. The rat was transcardially perfused with PBS (pH- 7.4) followed by 4% paraformaldehyde in PBS [26]. After perfusion the brains were dissected and immersion fixed in 4% paraformaldehyde for 1 hr and then equilibrated with 30% sucrose solution in 0.1 M PBS. 40 μm sections were cut using Cryostat (Leica, CM1510 S). The sections were treated with PBST (PBS in 0.05% Triton X-100) for 20 min. Brain slices were incubated overnight at 4°C with rat primary antibody for GABA_Aα_1. The brain slices were then rinsed with PBST and then incubated with Rhodamine coated secondary antibody. The sections were observed and photographed using confocal imaging system (Leica SP 5).

## Results

### Scatchard analysis of [^3^H]GABA binding against GABA in the cerebral cortex of Control, Epileptic, Epileptic + *Bacopa monnieri*, Epileptic + Bacoside A, and Epileptic + Carbamazepine treated rats

Scatchard analysis of [^3^H]GABA against GABA in the cerebral cortex showed a significant decrease (P < 0.001) in the B_max _of epileptic rats compared to controls. K_d _showed significant decrease (P < 0.05) in the epileptic group compared to control. Treatment using *Bacopa monnieri*, Bacocide-A and Carbamazepine reversed the B_max _to near control (Table 1).

**Table 1:** Scatchard analysis of [3H]GABA binding against GABA in the cerebral cortex of control and experimental rats

Group	B_max _(fmoles/mg ptn)	K_d _(nM)
C	112.3 ± 6.3	2.6 ± 0.3

E	44.0 ± 3.5***	1.5 ± 0.1**

E+B	89.8 ± 5.6^@@^	2.3 ± 0.2

E+D	93.2 ± 4.7^@@^	2.2 ± 02

E+C	80.7 ± 5.2^@@^	2.1 ± 0.1

Values are mean ± SEM of 4-6 separate experiments. Each group consist of 6-8 rats ** p < 0.01, *** p < 0.001 when compared to control, @@ < 0.01, @@@ < 0.001 when compared to epileptic group. C- Control, E- Epileptic, E+B- Epileptic rats treated with *Bacopa monnieri*, E+D- Epileptic rats treated with Bacoside A and E+C- Epileptic rats treated with Carbamazepine.

### Scatchard analysis of [^3^H]bicuculline against Bicuculline and [^3^H]baclofen against baclofen in the cerebral cortex of Control, Epileptic, Epileptic + *Bacopa monnieri*, Epileptic + Bacoside A and Epileptic + Carbamazepine, treated rats

Scatchard analysis of [^3^H]bicuculline against bicuculline and [^3^H]baclofen against baclofen in the cerebral cortex showed a significant decrease (P < 0.001) in the B_max _of epileptic rats compared to controls. K_d _showed significant decrease (P < 0.05) in epileptic group compared to control. Treatment using *Bacopa monnieri*, Bacocide-A and Carbamazepine reversed the B_max _to near control (Tables 2 and 3).

**Table 2:** Scatchard analysis of [^3^H]bicuculline binding against bicuculline in the cerebral cortex of control and experimental rats

Group	B_max _(fmoles/mg ptn)	K_d _(nM)
C	85.4 ± 4.5	1.7 ± 0.3

E	37.9 ± 3.9***	0.9 ± 0.1**

E+B	75.0 ± 5.3^@@@^	1.2 ± 0.3

E+D	80.1 ± 6.1^@@@^	1.39 ± 0.1

E+C	76.5 ± 4.7^@@@^	1.38 ± 0.2

Values are mean ± SEM of 4-6 separate experiments. Each group consist of 6-8 rats ** p < 0.01, *** p < 0.001 when compared to control, @@ < 0.01, @@@ < 0.001 when compared to epileptic group. C- Control, E- Epileptic, E+B- Epileptic rats treated with *Bacopa monnieri*, E+D- Epileptic rats treated with Bacoside A and E+C- Epileptic rats treated with Carbamazepine.

**Table 3:** Scatchard analysis of [^3^H]baclofen binding against Baclofen in the cerebral cortex of control and experimental rats

Group	B_max _(fmoles/mg ptn)	K_d _(nM)
C	124.3 ± 8.5	1.8 ± 0.2

E	91.9 ± 6.9***	1.2 ± 0.1**

E+B	115.2 ± 7.3^@@@^	1.6 ± 0.3

E+D	111.7 ± 5.1^@@@^	1.3 ± 0.1

E+C	107.1 ± 7.7^@@@^	1.4 ± 0.2

Values are mean ± SEM of 4-6 separate experiments. Each group consist of 6-8 rats ** p < 0.01, *** p < 0.001 when compared to control, @@ < 0.01, @@@ < 0.001 when compared to epileptic group. C- Control, E- Epileptic, E+B- Epileptic rats treated with *Bacopa monnieri*, E+D- Epileptic rats treated with Bacoside A and E+C- Epileptic rats treated with Carbamazepine.

### Real Time-PCR analysis of GABA_Aά1_, GABA_Aά5 _GABA_Aγ _and GABA_Aδ_, receptor subunit mRNA in the cerebral cortex of Control, Epileptic, Epileptic + *Bacopa monnier *, Epileptic + Bacoside A and Epileptic + Carbamazepine treated rats

**Figure 1:**
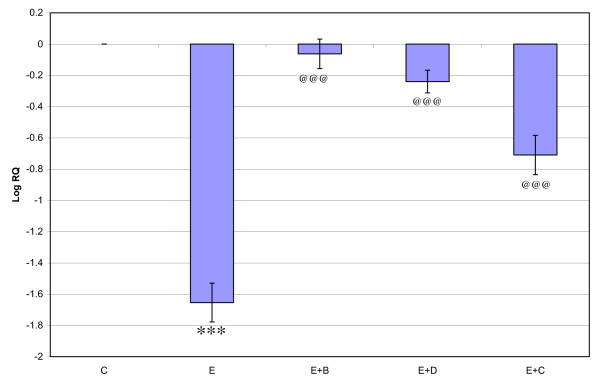
**Representative graph showing Real-Time amplification of GABA _Aα1 _receptor subunit mRNA from the Control and experimental rats**. The ΔΔCT method of relative quantification was used to determine the fold change in expression. Values are mean ± S.D of 4-6 separate experiments. C- Control, E- Epileptic, E+B- Epileptic + *Bacopa monnieri*, E+D- Epileptic + Bacoside-A, E+C- Epileptic + carbamazepine treated rats. ***p < 0.001 when compared to control, ^@@@^p < 0.001 when compared to epileptic group.

**Figure 2:**
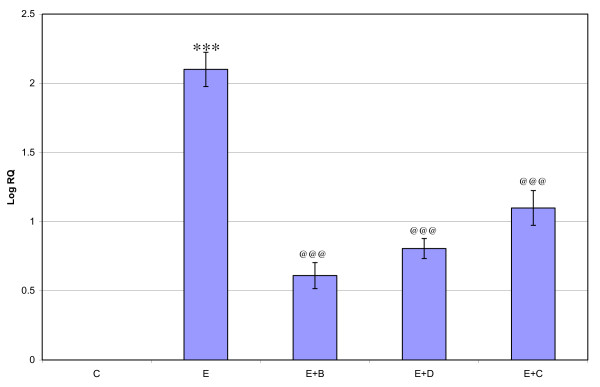
**Representative graph showing Real-Time amplification of GABA_Aα5 _receptor subunit mRNA from the Control and experimental rats**. The ΔΔCT method of relative quantification was used to determine the fold change in expression. Values are mean ± S.D of 4-6 separate experiments. C- Control, E- Epileptic, E+B- Epileptic + *Bacopa monnieri*, E+D- Epileptic + Bacoside-A, E+C- Epileptic + carbamazepine treated rats. ***p < 0.001 when compared to control, ^@@@^p < 0.001 when compared to epileptic group.

**Figure 3:**
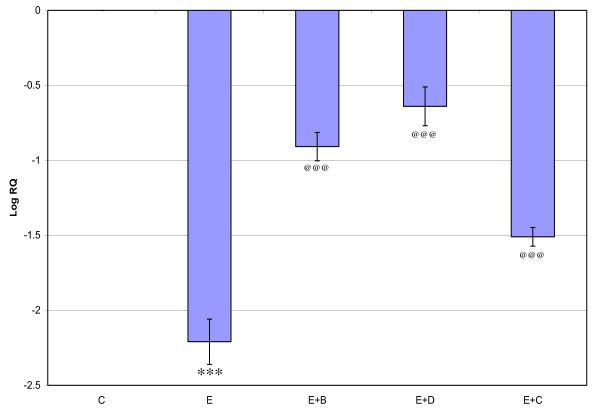
**Representative graph showing Real-Time amplification of GABA _Aγ5 _receptor subunit mRNA from the Control and experimental rats**. The ΔΔCT method of relative quantification was used to determine the fold change in expression. Values are mean ± S.D of 4-6 separate experiments. C- Control, E- Epileptic, E+B- Epileptic + *Bacopa monnieri*, E+D- Epileptic + Bacoside-A, E+C- Epileptic + carbamazepine treated rats. ***p < 0.001 when compared to control, ^@@@^p < 0.001 when compared to epileptic group.

**Figure 4:**
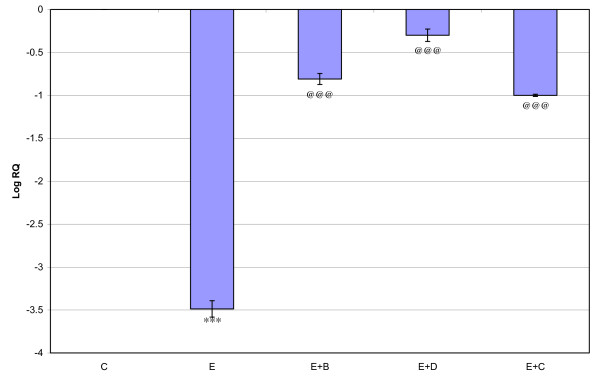
**Representative graph showing Real-Time amplification of GABA _Aδ5 _receptor subunit mRNA from the Control and experimental rats**. The ΔΔCT method of relative quantification was used to determine the fold change in expression. Values are mean ± S.D of 4-6 separate experiments. C- Control, E- Epileptic, E+B- Epileptic + *Bacopa monnieri*, E+D- Epileptic + Bacoside-A, E+C- Epileptic + carbamazepine treated rats. ***p < 0.001 when compared to control, ^@@@^p < 0.001 when compared to epileptic group.

Gene expression of GABA_Aά1_, GABA_Aγ _and GABA_Aδ_, where showed significant down regulation (p < 0.001) in the cerebral cortex of the epileptic rats compared to the control. Gene expression of GABA_Aά5 _receptor subunit showed significant up regulation compared to the control. Treatment using *Bacopa monnieri*, Bacocide-A and Carbamazepine reversed the changes to near control (Figures 1, 2, 3 and 4).

### Real Time-PCR analysis of GABA_B_, GAD65 and CREB mRNA in the cerebral cortex of Control, Epileptic, Epileptic + *Bacopa monnieri*, Epileptic + Bacoside A and Epileptic + Carbamazepine treated Epileptic rats

**Figure 5:**
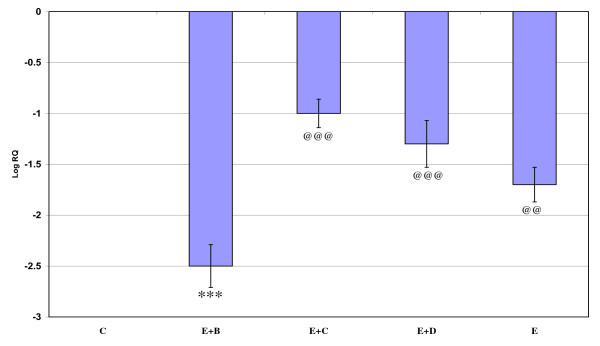
**Representative graph showing Real-Time amplification of GABA_B _receptor mRNA from the Control and experimental rats**. The ΔΔCT method of relative quantification was used to determine the fold change in expression. Values are mean ± S.D of 4-6 separate experiments. C- Control, E- Epileptic, E+B- Epileptic + *Bacopa monnieri*, E+D- Epileptic + Bacoside-A, E+C- Epileptic + carbamazepine treated rats. ***p < 0.001 when compared to control, ^@@@^p < 0.001 when compared to epileptic group.

**Figure 6:**
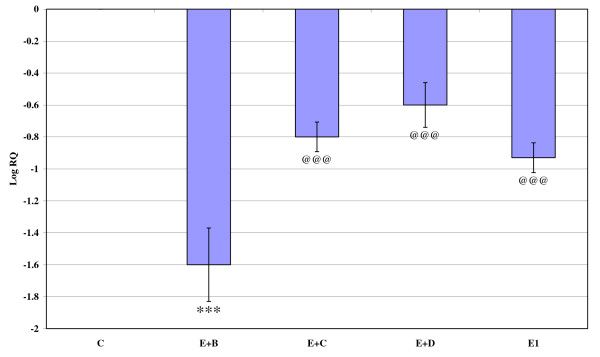
**Representative graph showing Real-Time amplification of GAD mRNA from the Control and experimental rats**. The ΔΔCT method of relative quantification was used to determine the fold change in expression. Values are mean ± S.D of 4-6 separate experiments. C- Control, E- Epileptic, E+B- Epileptic+ *Bacopa monnieri*, E+D- Epileptic + Bacoside-A, E+C- Epileptic + carbamazepine treated rats. ***p < 0.001 when compared to control, ^@@@^p < 0.001 when compared to epileptic group.

**Figure 7:**
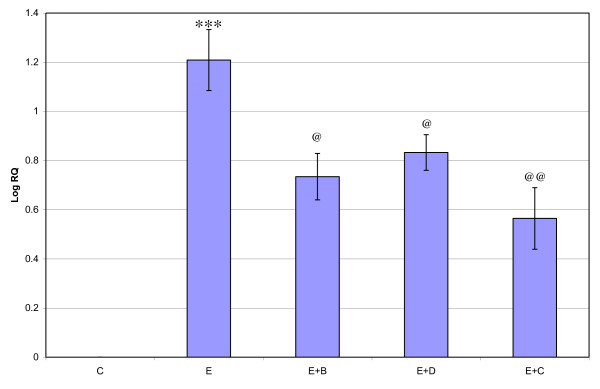
**Representative graph showing Real-Time amplification of CREB mRNA from the Control and experimental rats**. The ΔΔCT method of relative quantification was used to determine the fold change in expression. Values are mean ± S.D of 4-6 separate experiments. C- Control, E- Epileptic, E+B- Epileptic+ *Bacopa monnieri*, E+D- Epileptic + Bacoside-A, E+C- Epileptic + carbamazepine treated rats. ***p < 0.001 when compared to control, ^@@^p < 0.01, ^@^p < 0.05 when compared to epileptic group.

Gene expression of CREB *mRNA *showed significant up regulation (p < 0.001) in the cerebral cortex of the epileptic rats compared to the control. GABA_B_, and GAD65 mRNA were significantly down regulated compared to the control. Treatment using *Bacopa monnieri*, Bacoside-A and Carbamazepine were reversed the changes to near control (Figures 5, 6 and 7).

#### Eight-Arm Radial Maze Performance

**Figure 8:**
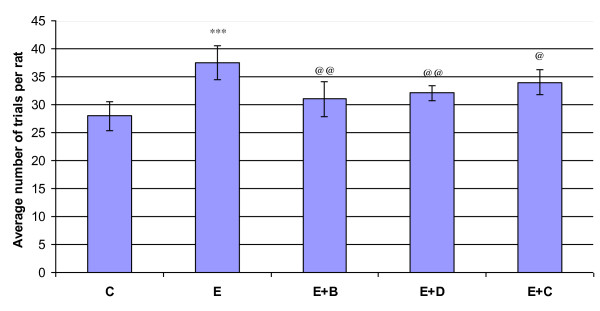
**Representative graph showing radial arm maze performance of control and experimental rats epileptic rats required more daily trials to achieve three, four, and five consecutive criterion performances**. Criterion performance was defined as consumption of the bait in the four baited arms of the radial maze during no more than five entries. There was no significant difference in the average number of trials required by kindled and control rats to achieve the initial criterion performance. C- Control, E- Epileptic, E+B- Epileptic + *Bacopa monnieri*, E+D- Epileptic + Bacoside-A, E+C- Epileptic + carbamazepine treated rats.

There was significant increase (p < 0.001) in the number of trials required to achieve five consecutive criterion performances in the epileptic rats compared to control. Treatment using *Bacopa monnieri *and Bacoside-A reversed this change to near control (Figure 8).

#### Y-Maze Performance

**Figure 9:**
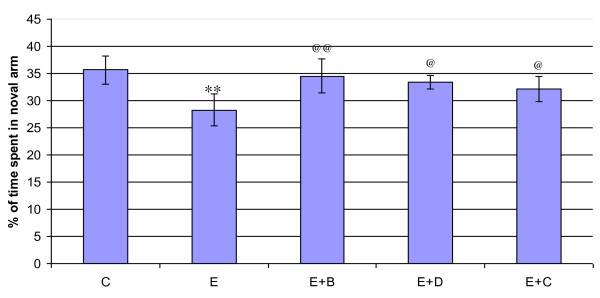
**Representative graph showing Y maze performance of control and experimental rats**. Epileptic rats showed less exploratory behavior compared to control. C- Control, E- Epileptic, E+B- Epileptic + *Bacopa monnieri*, E+D- Epileptic + Bacoside-A, E+C- Epileptic + carbamazepine treated rats. **p < 0.01 when compared to control, ^@@^p < 0.01 and ^@^p < 0.05 when compared to epileptic group.

Time spent in the novel arm was decreed significantly (p < 0.001) in the epileptic group compared to control. *Bacopa monnieri *and Bacocide-A treated epileptic rats were showed improved performance (Figure 9).

### GABA_Aα1 _receptor antibody staining in the Hippocampus of Control, Epileptic, Epileptic + *Bacopa monnieri*, Epileptic + Bacoside A and Epileptic + Carbamazepine treated Epileptic rats

**Figure 10:**
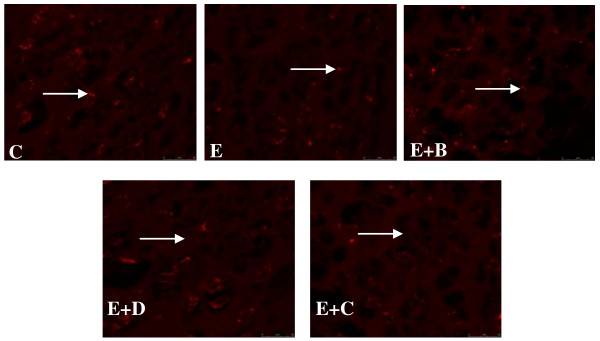
**GABA_Aα1 _receptor subunit antibody staining in the cerebral cortex of Control**. and experimental rats. C- Control, E- Epileptic, E+B- Epileptic + *Bacopa monnieri*, E+D- Epileptic + Bacoside-A, E+C- Epileptic + carbamazepine treated rats. Pixel intensity of experimental rats were C-103465 ± 3043, E- 76456 ± 1593**, E+B-95835^@@ ^± 2935, E+D-90564 ± 2553^@@^, E+C-87593 ± 1093^@@^. () Indicating the GABA_Aα1 _receptor subunit. **p < 0.01 when compared to control, ^@@^p < 0.01 when compared to epileptic group.

GABA_Aα1 _receptor subunit antibody staining in the hippocampus showed a significant decrease (p < 0.01) in the GABA_Aα1 _receptor subunit in epileptic rat compared to control. *Bacopa monnieri*, Bacoside-A and Carbamazepine treated epileptic rats showed a significant reversal (p < 0.05) of the decrease in GABA_Aα1 _receptor subunit staining in the hippocampus compared to epileptic rats (Figure 10).

## Discussion

In this study, we focused on the involvement of cortical GABA receptors in temporal lobe epilepsy, associated mood disorders, spatial learning and memory deficit in epileptic rats. TLE is a common end result of brain-damaging insults with very different etiologies and initial pathologies, such as genetic malformation, head trauma, stroke, infection or status epilepticus [27]. Although there are various reports of the anticonvulsant and neuroprotective properties of *Bacopa monnieri *and its active component Bacoside-A, little effort has been extended to understand their pharmacological action on the GABA receptors in the cerebral cortex of the epileptic rats. GABA is the major inhibitory neurotransmitter in the central nervous system [28,29]. It exerts an inhibitory action in all forebrain structures and plays a role in the physiopathogenesis of epilepsy [30]. GABA_A _receptor binding influences the early portion of the GABA mediated inhibitory postsynaptic potential, whereas GABA_B _binding influences the late portion. GABA_A _receptor activation in neurons induced a complex physiological response, namely the activation of a Cl^- ^conductance in concert with a blockade of the resting K^+ ^outward conductance results in hyperpolarisation. Both responses were mediated by the activation of GABA_A _receptors, since they were both mimicked by the GABA_A _receptor agonist muscimol and antagonized by picrotoxin and bicuculline [2]. Reductions of GABA mediated inhibition and decreased activity of GAD has been reported in studies of human epileptic brain tissue. Impairment of GABA functions produces seizures, whereas enhancement results in an anticonvulsant effect [31,32]. Support for a chronic loss of GABAergic function in epileptogenic human cortex has been based on biochemical assays of tissue resected for relief of focal seizure [33,34]. It is reported that in both epileptic and histologically damaged cortex, there are significant decreases in GAD and GABA binding [33]. We observed a significant decrease in the B_max _of GABA receptors in the cerebral cortex of epileptic rats compared to control GABA receptors and GAD gene expression patterns were similar to the receptor binding studies. Treatment with *Bacopa monnieri *and Bacoside-A reversed the receptor alterations in B_max _and gene expression to near control. Previous studies from our lab showed that *Bacopa monnieri *treatment to epileptic rats reduced the number of seizures per hour which is suggestive of its anticonvulsant property [35]. GAD plays a very important role in maintaining excitatory inhibitory balance of the central nervous system [36]. Analysis of GAD activity was used as a marker of over all GABAergic activity and was found to be lower in epileptic mice. The enzymatic activity of GAD is the rate limiting step in the production of GABA and GAD serve as a marker of inhibitory neurons. Moreover, preliminary findings indicate that the decrease in GABA is associated with reduced GABA synthesis rather than increased degradation [37]. In order to provide a direct anatomic analysis of GABAergic neurons and terminals in experimental epilepsy, it is demonstrated a significant decrease in immunocytochemically labeled GAD-positive neurons and puncta in and adjacent to an alumina-damaged epileptic focus in monkey motor cortex [38-40].

There are seizure-dependent increases in CRE-binding activities in various brain regions of the mice [41]. Our study showed that CREB mRNA was significantly decreasing in the cerebral cortex of the epileptic rats. Increased expression of total CREB and phosphorelated CREB found in the human epileptic hippocampus. This when considered with the observation that CREB mediate transcription from the GABA_B _Receptor1b (GABA_B_R1_b) _and GABA_B_R1_b _promoters [42]; epileptic rats exhibit elevated levels of GABA B Receptor 2 (GABA_B_Rs) subunit mRNAs and GABA_B_Rs antagonist inhibit CREB-DNA binding activity at dose that inhibit seizure behaviour, point to a novel feed back mechanism to GABA_B _receptor in the generation and propagation of seizure.

The incidence of psychiatric diseases in epileptics is significantly higher than in the general population [43-46]. Depressive and anxiety disorders are the most common psychiatric diseases in this patient group. Recent studies employing magnetic resonance spectroscopy (MRS) suggest that unipolar depression is associated with reductions in cortical GABA levels [47,48]. Antidepressant and mood-stabilizing treatments also appear to raise cortical GABA levels and to ameliorate GABA deficits in patients with mood disorders [49]. Anxiety disorders have long been associated with disturbances of GABA function because of the ability of the benzodiazepine anxiolytics to facilitate brain GABA neurotransmission [50]. Interestingly, as with plasma studies MRS also reveals lowered concentrations of GABA in occipital cortex in panic disorder [51] and in subjects with alcohol dependence [52]. We used two validated psychopharmacological method Y-maze test to quantify depression-like and anxiety-like behavior induced by the decreased GABA receptors in the cerebral cortex of the epileptic rats. Our study showed decreased exploratory behaviour in epileptic rats and treatment using *Bacopa monnieri *and bacoside-A brings back the exploratory behaviour to near control. This can be correlated with the decreased GABA receptors in the cerebral cortex of the epileptic rat interaction through 5-HT pathways. Immobility in rats is considered to be a state of lowed mood or hopelessness which the rodent experience when they are forced to explores in a constrained space from they cannot escape. There are multiple interactions between central GABA and 5-HT pathways, and some of these interactions provide a theoretical framework in which changes in cortical GABA function can lead to some aspects of disrupted 5-HT function seen in depressed patients [53,54]. In addition, interactions between 5-HT and GABA offer a route by which 5-HT targeted treatments might alter GABA function to bring about their therapeutic effect [55]. Nigel et al., [56] have suggested shared neurobiological processes leading both to seizures and to behavioral, emotional and cognitive disturbance which could possibly explain the how *Bacopa monnieri *is effective as an anti-convulsant and an anti-depressant. Carbamzepine treatment to epileptic rats also showed a similar effect.

Cerebral cortex plays a key role in memory, attention, perceptual awareness, thought, language, mood and consciousness. Epileptic patients are often suffering from memory and cognitive problems [57-59]. Preliminary studies established that the treatment with crude extract and with the alcoholic extract of *Bacopa monnieri *plant [60] enhanced learning ability in rats. The radial arm maze experiment was conducted to study the neurobiological mechanisms that underlie spatial learning and memory functions in experimental. Radial arm maze is a tool to examine the neural systems that are involved in memory, and the influence of pharmacological compounds on memory [61]. Radial arm maze experiment demonstrated the impairment in spatial learning and memory in the model studied. The administration of a crude extract of *Bacopa monnieri *to epileptic rats decreased the trial to attain the criterion performance in the radial arm maze to control levels. These results confirmed the memory enhancing property of *Bacopa monnieri *in epileptic rats. It is also reported to facilitate the acquisition, consolidation, retention and recall of learned tasks [62] and improve the speed at which visual information is processed. *Bacopa monnieri *extracts and isolated bacosides have been extensively investigated in several studies for their neuropharmacological effects and a number of reports are available confirming their neuroprotective action [35].

## Conclusion

Our experimental results support that decreased GABA receptors and GAD activity in the cerebral cortex comprise an important role in seizure initiation and mood disorders associated with epilepsy. We conclude from our studies that *Bacopa monnieri *and Bacoside-A treatment potentiates a therapeutic effect by reversing the alterations in general GABA, GABA_A_, GABA_B _receptor binding, GABA_A _receptor subunits, GAD and CREB gene expression that occur during epilepsy, resulting an increased GABA mediated inhibition in the over stimulated cerebral cortex neurons. *Bacopa monnieri *and Bacoside-A treatment also useful for managing the memory problems and mood disorders associated with the epilepsy. This studies showed the therapeutic significance of *Bacopa monnieri*, and its active component Bacocide-A in the management of epilepsy, associated mood disorders and memory problems.

## Competing interests

The authors declare that they have no competing interests.

## Authors' contributions

JM designed the work and carried out the receptor study. SB participated in the gene amplification studies. SAP and PMA participated in the co focal and behavioural studies. All authors read and approved the final manuscript.
